# Two Methods of Forward Head Posture Assessment: Radiography vs. Posture and Their Clinical Comparison

**DOI:** 10.3390/jcm13072149

**Published:** 2024-04-08

**Authors:** Paul A. Oakley, Ibrahim M. Moustafa, Jason W. Haas, Joseph W. Betz, Deed E. Harrison

**Affiliations:** 1 CBP Nonprofit (A Spine Research Foundation), Eagle, ID 83616, USA; docoakley.icc@gmail.com (P.A.O.); drjasonhaas@gmail.com (J.W.H.); drjoebetz@gmail.com (J.W.B.); 2 Private Practice, Newmarket, ON L3Y 8Y8, Canada; 3 Kinesiology and Health Science, York University, Toronto, ON M3J 1P3, Canada; 4 Department of Physiotherapy, College of Health Sciences, University of Sharjah, Sharjah 27272, United Arab Emirates; iabuamr@sharjah.ac.ae; 5 Neuromusculoskeletal Rehabilitation Research Group, RIMHS–Research Institute of Medical and Health Sciences, University of Sharjah, Sharjah 27272, United Arab Emirates; 6 Private Practice, Boise, ID 83709, USA

**Keywords:** neck pain, craniovertebral angle, forward head posture, X-ray, cervical spine, sagittal balance

## Abstract

**Background:** Forward head posture (FHP) and altered cervical lordotic curvatures are common spine displacements often associated with neck pain and disability. Two primary categories for determining FHP exist: radiographic and postural measurements. **Methods:** This study investigated the correlation between the craniovertebral angle (CVA), the radiographically measured C2–C7 sagittal vertical axis (SVA), and cervical lordosis (absolute rotation angle: ARA C2–C7) in a sample of participants with chronic myofascial pain (CMP). In 120 participants, we performed both a postural measurement of the CVA and a lateral cervical radiograph, where the C2–C7 SVA and ARA C2–C7 were measured. A linear-regression R^2^ value to assess the correlation between the CVA, C2–C7 SVA, and ARA C2–C7 was sought. **Results:** A statistically significant weak linear fit was identified (Spearman’s r = 0.549; R^2^ = 0.30, *p* < 0.001) between the CVA and C2–C7 SVA, having considerable variation between the two measures. A statistically significant linear fit (very weak) was identified for the lordosis ARA C2–C7 and the CVA: Spearman’s r = 0.524; R^2^ = 0.275; *p* < 0.001. A value of 50° for the CVA corresponded to a value of 20 mm for the C2–C7 SVA on an X-ray. **Conclusion:** While the CVA and radiographic C2–C7 SVA are weakly correlated in an individual, they seem to represent different aspects of sagittal cervical balance. The CVA cannot replace radiographically measured cervical lordosis. We recommend that more emphasis be given to radiographic measures of sagittal cervical alignment than the CVA when considering patient interventions.

## 1. Introduction

Forward head posture (FHP) is one of the most common postural displacements and is estimated to be of a clinically relevant magnitude in two-thirds of the human patient population [1,2,3]. Studies have found that there is a significant association between neck pain and forward head posture, with higher risks of having neck pain in female and older populations [3]. It is generally believed that this abnormal posture is associated with the development and persistence of spine pain and various biomechanically driven disorders [4,5,6,7,8,9,10]. For example, researchers have identified that FHP alters the cervical range of motion (ROM) [4], contributes to an abnormal balance [5,6], and alters respiratory efficiency [7]. It has been readily seen in the past decade that there has been a surge in interest in forward head posture in surgical [8,9] and conservative rehabilitation settings alike [3].

Two primary categories for determining FHP exist: radiographic assessments and external postural measurements. Harrison and colleagues [10,11,12] originally presented a radiographically determined measurement of FHP using the C2–C7 sagittal plumbline, and, later, in 2012, this was modified by Tang et al. [13]; both these radiographic methods are in use today [14,15]. In terms of external measurements of FHP, there are a variety of methods [15,16]. A very common method used is the craniovertebral angle (CVA) [1,2,3,4,5,6,7,16,17]. In reviewing the literature, it becomes apparent that, in the surgical setting, radiographic measurements are favored [8,9,10,13,15], while, in the conservative rehabilitation literature, external postural measurements are emphasized [1,2,3,4,5,6,7,16,17]. However, there are exceptions where some conservative care researchers prefer radiographic methods to assess sagittal cervical alignment [14,17,18,19]. Arguably, the use of external vs. internal measurement methods in attempting to quantify FHP may create conflicting understandings, interventions, and outcomes.

A radiographic analysis of the sagittal cervical spine also provides the essential component of the assessment of cervical lordotic curvature or lack thereof [9,10,11,12,13,14,15]. While there is an inherent relationship between FHP and cervical lordotic curvature due to kinematic effects, this correlation may only be significant for larger or end-range movement postures [8]. Recently, in the surgical literature, the magnitude of the C2–C7 cervical lordotic curvature relative to the slope of the T1 vertebra has been postulated as an important variable affecting pain, disability, and generalized poor outcomes [9]. Similarly, in the conservative rehabilitation literature, a C2–C7 cervical lordosis of 20° has been found to be a statistically significant magnitude that is associated with improved patient outcomes in randomized trials [18,19]. Moustafa and colleagues, for example, identified that the improvement in cervical lordosis coupled with the reduction in FHP in a radiographic analysis was linearly correlated with an improved central conduction time (a neuro-physiological measure of spinal cord velocity under somato-sensory-evoked potentials) [18].

Given the significance of external measurements of FHP, radiographically measured C2–C7 translation, and the amount of cervical lordosis, an understanding of how external postural measures correlate with radiographic sagittal cervical alignment would be of considerable importance. In this regard, we located only one investigation that compared radiographically measured CVA to postural photographs measuring the CVA in a sample of 40 people [20]; this lack of information in the literature is likely a result of fear of risk posed by ionization radiation exposure. This investigation [20] used seated positioning and markers in place with both the photographic and radiographic measurement methods, and a good correlation (Pearson’s r = 0.89) was identified between the two methods for CVA measurement. However, we identified no study that has compared the correlation between the most common method (the CVA) of postural measurement of FHP to that of the C2–C7 radiographically determined sagittal vertical axis (SVA). Accordingly, our study seeks to investigate the correlation between the postural CVA and the radiographically measured C2–C7 SVA in a sample of participants with chronic myofascial pain (CMP).

Our investigation uses a sample of 120 participants with chronic myofascial pain (CMP) who were part of a previously published trial [21], who had undergone a postural measurement of the CVA and a lateral cervical radiograph where their C2–C7 SVA had been assessed. Our study hypothesis is that the external posture measurement using the CVA will have a moderate-to-strong linear correlation to the radiographic C2–C7 SVA, such that these two can be used interchangeably in individual patients. Our second hypothesis is that the CVA will also have a moderate-to-strong linear correlation to cervical lordosis.

## 2. Materials and Methods

The current investigation utilizes data collected previously from a prospective, investigator-blinded, parallel-group, randomized, controlled trial [21]. The trial was registered with the Clinical Trial Registry {PACTR201801002968301}. Our university’s institutional review board approval was obtained prior to this study; all the participants signed their informed consent and were recruited from our institution’s local outpatient clinic. Patients with cervical CMP were recruited from the university’s rehabilitation clinic from March 2016 to October 2017.

### 2.1. Participants

The participants were screened prior to inclusion for alterations in two primary cervical alignment variables: loss of the cervical lordosis and anterior head translation. Participants were included (1) if their cervical lordosis was between 0° and 20°, as measured using the intersection of two lines drawn along the posterior body margins of C2 and C7, and (2) if their radiographically measured C2–C7 SVA was ≥15 mm [21]. Concerning the photographically measured FHP, the participants had to have a significant anterior head translation as measured by the CVA. If the CVA was less than or equal to 50°, then a participant was referred to this study. Our selection of 50° as a reference angle was guided by the study of Yip et al. [22]. For this randomized trial, 120 participants met the inclusion criteria, and these participants’ data have been utilized in our current investigation. Participants were excluded if any signs or symptoms of medical “red flags” were present: tumor, fracture, rheumatoid arthritis, osteoporosis, and prolonged steroid use. Additionally, participants were excluded based on any previous spinal surgery and any exam findings consistent with neurological diseases and vascular disorders.

### 2.2. Outcome Assessments

All outcome assessments were carried out with the same data collectors to prevent potential recorder and ascertainment biases. Two measurements of FHP were collected for each of the 120 participants: (1) the CVA (°) and (2) the radiographic C2–C7 SVA (mm).

#### 2.2.1. Forward Head Posture (FHP) Assessment with the Craniovertebral Angle (CVA)

**Figure 1:**
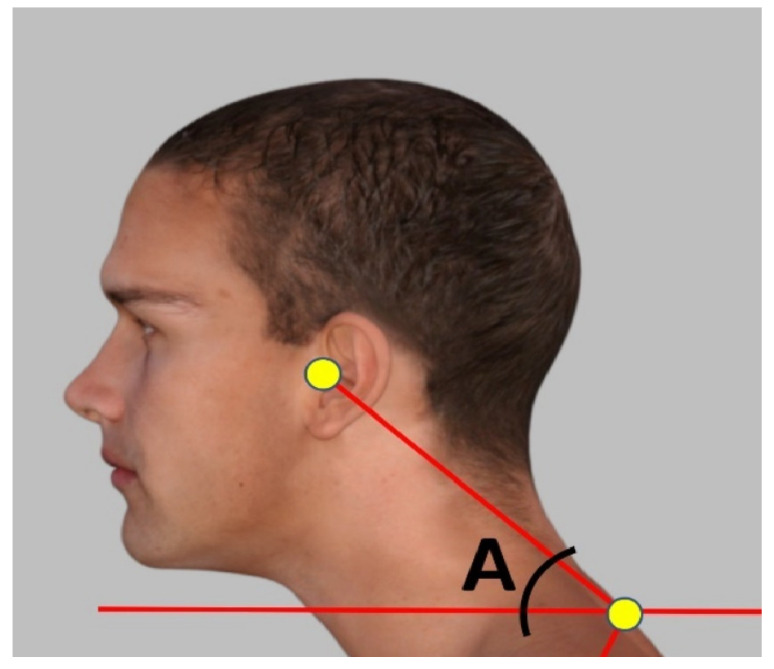
The craniovertebral angle (CVA). Adhesive marker placement locations at the C7 spinous process and the tragus of the ear. A line is constructed between the two markers, and then a horizontal line is drawn through the C7 marker; angle A is the CVA. This method has excellent examiner reliability [16,17].

The assessment of the FHP was carried out by measuring the craniovertebral angle. Standing cervical posture alignment was measured with photogrammetry, which provides a valid and reliable indicator of forward head posture using the CVA [16,17,23,24]. Figure 1 demonstrates the measurement of the CVA. If the angle was less than 50 degrees, it was considered as FHP [22]. The measurement technique was duplicated, like in the study by Diab and Moustafa [25], as follows: Adhesive markers (8 mm diameter) were placed on a participant’s C7 spinous process and tragus of the ear. The therapist observed the participant from the lateral side while standing and then took a picture of the participant from a fixed distance (75 cm) and height (150 cm); the angle was measured by placing each vector as if it were following a line from the tragus of the ear to the C7 spinous process and another horizontal line through the C7 spinous process [25]. Thus, the CVA was the angle formed at the intersection between a horizontal line through the spinous process of C7 and a line to the tragus of the ear.

#### 2.2.2. Lateral Cervical Radiography: ARA C2–C7 and C2–C7 SVA

**Figure 2:**
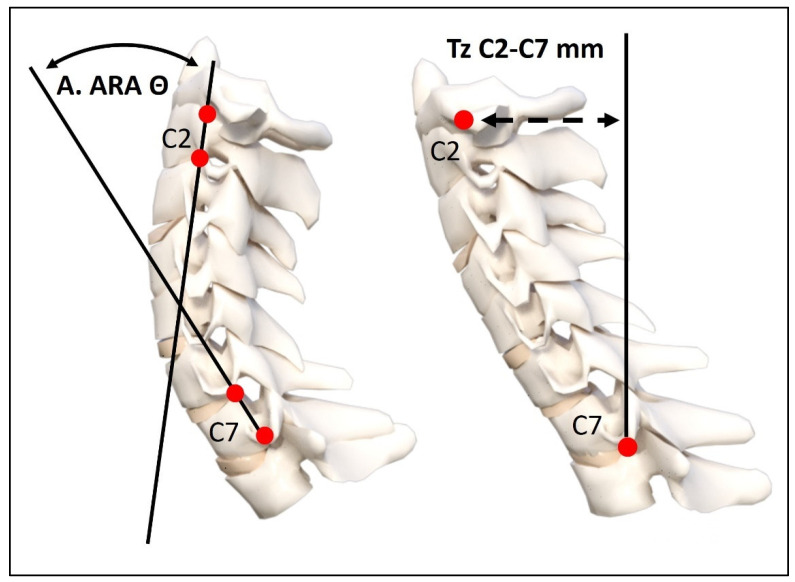
The ARA C2–C7 lordosis measurement and the C2–C7 SVA [11]. ARA C2–C7 is formed by drawing lines along the posterior body margins of C2 and C7 and measuring their angle of intersection. For the C2–C7 SVA, a vertical line originates at the posterior inferior corner of cervical vertebra C7, and the horizontal distance of the posterior superior body corner of cervical vertebra C2 is measured. Currently, this has been modified and adopted in the surgical literature as the sagittal vertical axis (SVA) of C2–C7, with a slight modification in the locations of the origins of the boney landmarks of C2 and C7 [9,13]. Both methods have excellent examiner reliability [11,16,26].

A standing lateral cervical spine radiograph was obtained with the participant in a relaxed neutral standing posture, with their right side against the X-ray cabinet. The radiographs were assessed for two different variables: (1) the absolute rotation angle (ARA) of cervical lordosis from C2 to C7, and (2) a radiographic FHP measurement of the horizontal displacement of vertebra C2 relative to C7. The ARA C2–C7 was formed by drawing a line along the posterior body margin of C2 and measuring the angle of the intersection with a second line drawn along the posterior body margin of C7. Secondly, the radiographic C2–C7 SVA was measured by assessing the horizontal offset of the posterior superior body corner of cervical vertebra number 2 relative to a vertical line extending further, originating from the posterior inferior body corner of cervical vertebra number 7. The C2–C7 SVA measurement method was originally developed by Harrison in 1982 [10] and is shown in Figure 2. In the orthopedic literature, this measurement is currently referred to as the SVA of C2–C7 [9,13,15]. The ARA C2–C7 lordosis method is often referred to as the Harrison posterior tangent method [12,16,26] as Harrison [10,11,12] was the first to apply this measurement method to the entire sagittal plane of the spine (C2-S1-inclusive) and the first to report normative data for the ARA C2–C7 [10]. In the orthopedic literature, some authors refer to this as the Gore method of cervical lordosis [27] (in 1986, Gore reported normative data in an asymptomatic population [28]). Both the ARA C2–C7 and the C2–C7 SVA have been found to have excellent intra- and inter-examiner reliability (ICC’s > 0.7), with small standard errors of measurement: <1–2 mm and <1–2° [11,26].

### 2.3. Data Analysis

Patient descriptive data and the radiographic variables are reported as means and standard deviations. Patient data were initially imported into Microsoft Excel (2018 Microsoft Excel, retrieved from https://office.microsoft.com/excel, accessed on 22 March 2024), and a statistical analysis was performed with SPSS (SPSS Inc. v.29, Chicago, IL, USA). To determine the normality of the collected numerical variables, the Shapiro–Wilk test was used. Since all three variables of interest were not normally distributed, Spearman’s ranked correlation coefficient (r) was used to investigate the correlations between the variables. Finally, the R^2^ linear regression model was used to compare the two FHP variables and the cervical lordosis variable to one another to determine the statistical fit and percentage variation between the two measurement methods for the assessment of FHP. Regression equations for the values of CVA were reported to predict the corresponding value for C2–C7 SVA. Also, regression equations predicting the ARA from both the CVA and C2–C7 SVA were completed. The level of significance was set to 0.05, and any correlation was considered statistically significant when the *p*-value < 0.05. A sample size determination for simple linear regression with a single predictor was calculated for a power level of 0.8, a significance level of 0.5, and a medium effect size. The required sample was determined to be 54 [https://www.statskingdom.com/sample_size_regression.html, accessed on 22 March 2024]; thus, our convenience sample (*n* = 120), used to perform our secondary analysis, was determined to be more than sufficient.

## 3. Results

### 3.1. Patient Demographics

One hundred and twenty patients (76 males) with chronic myofascial pain syndrome (MPS) were included in this investigation. The demographic characteristics of the patients are shown in Table 1. Our population had CMP, with an average neck pain intensity of 5.2/10 on the numerical rating scale (0 = no pain; 10 = worst pain ever). The AHT C2–C7 averaged 2.46 cm, with a maximum of 3.7 cm and a minimum of 1.5 cm. The ARA C2–C7 averaged 6.8, with a maximum value of 0° (straightened curve) and a minimum value of −17° lordosis. The CVA averaged 44.9°, with a maximum value of 51° and a minimum value of 40°.

**Figure 3:**
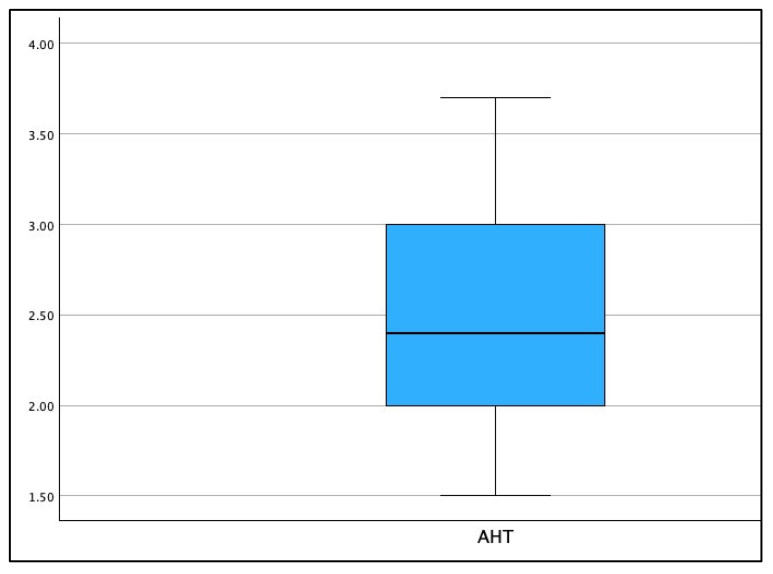
Boxplot of the distribution for radiographically measured anterior head translation (AHT) C2–C7 (cm) in the population of 120 patients.

**Figure 4:**
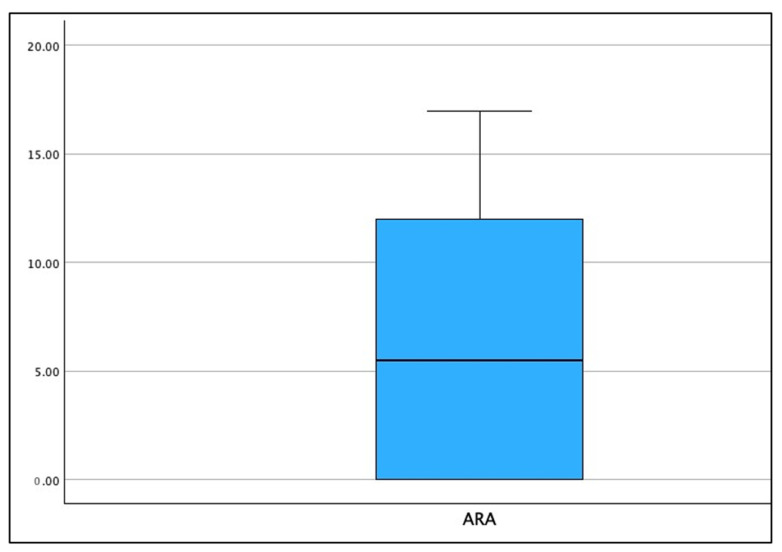
Boxplot of the distribution for radiographically measured cervical lordosis absolute rotation angle (ARA) C2–C7 (°) in the population of 120 patients. The absolute value is presented where lordosis is a positive number in this plot. Note, there is no lower ‘whisker’, as the inclusion criteria included lordosis values between 0 and 20°.

**Figure 5:**
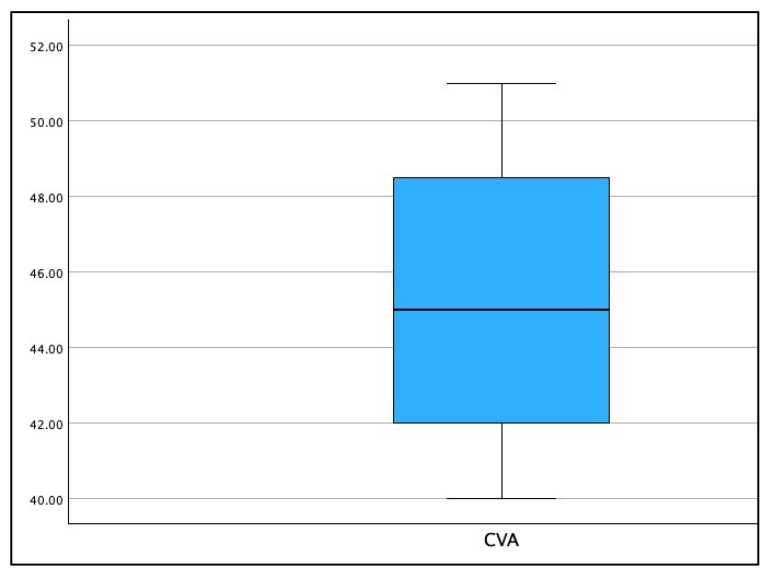
Boxplot of the distribution for the forward head posture as measured with the craniovertebral angle CVA (°) in the population of 120 patients.

Figure 3, Figure 4 and Figure 5 show the boxplot for the distributions for AHT C2–C7, ARA C2–C7, and CVA, respectively. It is noted that a boxplot visually shows the distribution of numerical data and their skewness by displaying the data in quartiles. The (thick) line within the box represents the median value, and the top and bottom of the box represents the 75th (Q3) and 25th (Q1) percentiles, respectively. The upper and lower ‘whiskers’ represent the 1.5 × interquartile range (Q3–Q1): that is, Q3 + 1.5 × IQR and Q1 − 1.5 × IQR, respectively.

### 3.2. Regression Outcome

**Figure 6:**
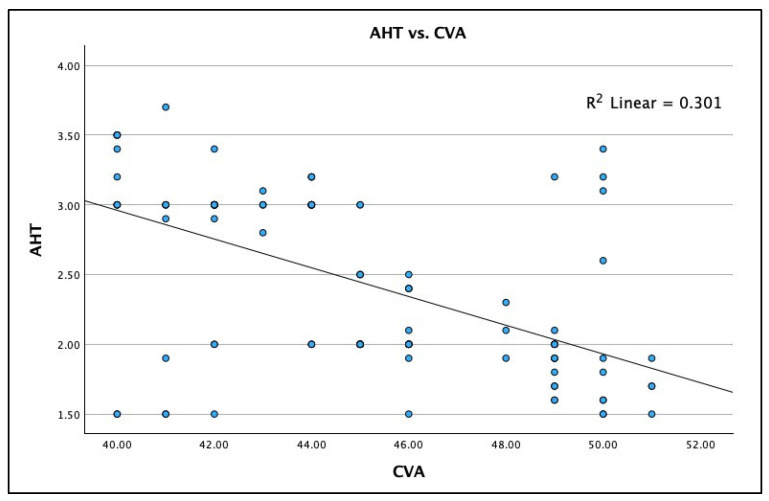
Linear regression correlation between the craniocervical angle (CVA) and the anterior head translation (AHT) C2–C7 sagittal vertical axis (SVA) in 120 participants with chronic myofascial pain. Linear regression results: (1) equation is: y = 7.08 − 0.1x (2) Spearman’s r = −0.549; (3) R^2^ = 0.301, *p* < 0.001.

A statistically significant negative linear fit (weak effect) was identified for the radiographically measured C2–C7 SVA versus the photographic measured CVA: Spearman’s r = −0.549; R^2^ = 0.301; and *p* < 0.001. Figure 6 shows this analysis as a scatterplot with the regression analysis. There is considerable variation between the two measurements of FHP in each person. As an approximation, a value of 50° for the CVA corresponds to a value of 2 cm (20 mm) for the C2–C7 SVA on an X-ray, both of which are approximate cutoff points known to be associated with a greater frequency of neck pain [22,29]. In some cases, the CVA undervalues the C2–C7 SVA while in others it overvalues it.

**Figure 7:**
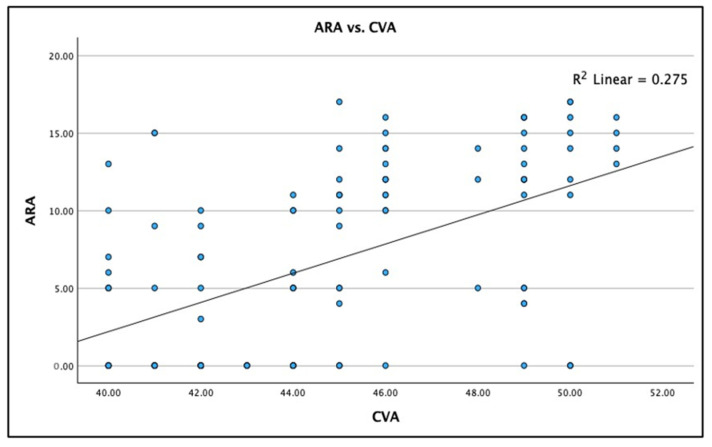
Linear regression correlation between the craniocervical angle (CVA) and the absolute rotation angle (ARA) C2–C7 in 120 participants with chronic myofascial pain. Linear regression results: (1) equation is CVA to predict ARA: y = −35.48 + 0.94x (2) Spearman’s r = 0.524; and (3) R^2^ = 0.275, *p* < 0.001.

A statistically significant linear fit (very weak effect) was identified for the radiographically measured cervical lordosis ARA C2–C7 versus the photographic measured CVA: Spearman’s r = 0.524; R^2^ = 0.275; and *p* < 0.001. Figure 7 shows this analysis as a scatterplot with the regression analysis. Though there is a very weak trend where increases in the CVA are associated with an increase in cervical lordosis (ARA C2–C7) there is large variation between the two measurements indicating that the CVA cannot be interchanged with the ARA C2–C7 lordosis in each person.

**Figure 8:**
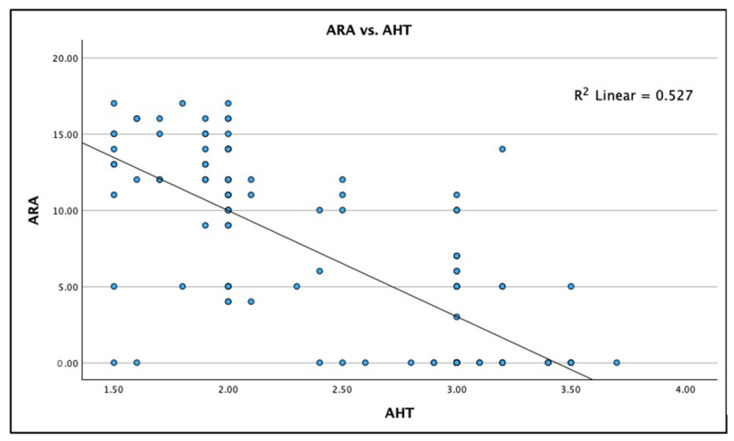
Linear regression correlation between the anterior head translation (AHT) C2–C7 sagittal vertical axis (SVA) and the absolute rotation angle (ARA) C2–C7 in 120 participants with chronic myofascial pain. Linear regression results: (1) equation is AHT to predict ARA: y = 23.87 − 6.95x (2) Spearman’s r = −0.726; and (3) R^2^ = 0.527, *p* < 0.001.

Lastly, there is a statistically significant linear fit (moderate effect) for the radiographically measured cervical lordosis ARA C2–C7 versus the radiographically measured C2–C7 SVA: Spearman’s r = −0.726; R^2^ = 0.527; and *p* < 0.001. Figure 8 shows this analysis as a scatterplot with the regression analysis. Though there is a moderate trend where increases in the C2–C7 SVA are associated with a straightening in cervical lordosis (ARA C2–C7), there is considerable variation between the two measurements, indicating that the C2–C7 SVA cannot be interchanged with the ARA C2–C7 lordosis in each person.

## 4. Discussion

The current investigation presents data collected from a previously published randomized trial investigating sagittal cervical treatment outcomes in a population of 120 participants with CMP syndrome [21], evaluated uniquely compared to the prior studies. In this trial, data from posture photographs measuring forward head posture (FHP) using the CVA were obtained for each participant along with lateral cervical (LC) radiographs measuring the C2–C7 SVA (plumb line translation) and the cervical lordosis ARA C2–C7 (angular measurement). The current investigation is the first, to our knowledge, to compare the CVA external posture measurement to the radiographic C2–C7 SVA and cervical lordosis in a population. We had hypothesized that there would be a moderate-to-strong correlation between the two measurements of a person’s FHP. Our results indicate that we must accept the opposite: namely, there is considerable variability between the CVA and the C2–C7 SVA. Further, we hypothesized that there would be a moderate-to-strong correlation between the CVA and cervical lordosis; however, we determined that the CVA is not associated with lordosis magnitude (ARA C2–C7), and, again, we must accept the contrary hypothesis. Thus, our data indicate that the cervical spine radiographic alignment of C2–C7 SVA and ARA cannot be simply exchanged for the CVA measured using postural photographs.

The current investigation used a linear regression analysis with the R^2^ model value to represent the proportion of variance (in %) in the measurements obtained for the CVA to predict the radiographic C2-C7 SVA, and for both the CVA and C2-C7 SVA to predict the ARA lordosis. In general, interpreting the relative strength of a relationship based on its R^2^ value is as follows: (1) none or very weak effect size R^2^ < 0.3; (2) a weak effect size 0.3 < R^2^ < 0.5; (3) a moderate effect size 0.5 < R^2^ < 0.7; and (4) a strong effect size R^2^ > 0.7 [30]. Accordingly, our results indicate a weak effect size (R^2^ = 0.3) for the comparison of radiographic C2–C7 SVA vs. the posture CVA measurement; that is, only 30% of the variance in radiographic FHP is shared with photographic FHP measures. Similarly, our results indicate a very-weak-to-no effect (R^2^ = 0.275) when looking at the correlation between the CVA and the cervical lordosis C2–C7.

Our investigation adds to the literature by determining the most appropriate and clinically relevant assessment for the determination of the FHP in an individual: namely, we argue that a sectional LC radiograph is the more important assessment to determine sagittal cervical spine alignment and caution should be used when interpreting a similar variable on a postural photograph. Having knowledge of this information should assist treatment recommendations for neck pain and other spine conditions, and our findings add validity to the necessity for both LC radiographs and postural photographs to accurately assess FHP. A clear presentation of our findings and a comparison to the existing literature follows.

### 4.1. Pros and Cons of the CVA

A comparison of radiography and postural images must include a discussion of their convenience of use, cost, and feasibility. The CVA is easier to obtain, does not require specialized licensure or physician supervision, and can be performed with any digital camera and a measurement APP [31]. The cost difference is significant, with the current costs for modern digital X-ray approaching $50,000 USD. This cost is often a factor in physicians choosing not to use radiography. Additionally, the higher cost of radiography vs. postural photographs is very appealing to third-party payors who are often looking at the financial aspect of treatment. The CVA has been studied and used by researchers and physicians for many years. The CVA is used by the human resources departments of corporations and researchers to determine proper ergonomics, and it is a simple tool [32,33]. Additionally, simple mobile devices such as Posture Screen^®^ (PostureCo^®^ Trinity, FL, USA) have been used across many fields to assess posture parameters with a cellphone [34,35,36]. Standard values have been obtained, and individuals with a CVA of less than 50 would be considered abnormal and would indicate a large FHP [22]. CVA angles measuring 55 degrees and greater would be considered more ideal, i.e., standard, as they indicate less FHP [3,15,16,22,23,37].

This convenience of use and absence of X-ray radiation has led many physicians, researchers, and institutional review boards to recommend and use posture image CVA in lieu of radiography for “patient safety” or “ethical considerations” to prevent the exposure of participants to ionizing radiation [3,16,22,23,24,38,39]. Although these “safety procedures” may be well intentioned, the results of this study demonstrate that the use of a postural image to assess CVA will be wrong 70% of the time compared to an X-ray measurement of C2–C7 SVA and lordosis (i.e., 1 − R^2^ = 1 − 0.3 = 0.7). Authors such as Cote et al. [38] and Mylonas et al. [16] concluded that telehealth and postural image analysis and surface contour should suffice for physicians to make treatment recommendations. This is counter to the data found in our study and the long known understanding that radiography is the gold standard in the treatment of spine pain and various other conditions [40,41,42]. Considering this evidence, the CVA and posture assessed by photos are insufficient for a physician to reach a conclusion regarding triage, diagnosis, treatment recommendations, and referral recommendations for any spinal pain and associated conditions. The CVA is an important adjunct diagnostic tool for postural measurements, and wise clinicians will use simple and inexpensive tools to assist them in a diagnosis; however, based on the current findings, the CVA should not replace radiography in clinical practice.

In circumstances where radiography is not available due to geographic limitations, socioeconomic limitations, or other factors, the CVA could be a valuable tool. Therapeutic providers and interventionists such as Yoga practitioners, Pilates instructors, exercise personal trainers, occupational and physical therapists, sports medicine providers, and human resources department evaluators could use the CVA for recommendations of postural correction with simple exercises designed to improve neck musculature and stability and posture. There is some evidence regarding a reduction in abnormal posture with postural exercises targeting neck muscles [43,44]. However, these practitioners and trainers should use extreme caution in recommendations of postural exercises for patients reporting pain or previous injury. This is because, in the absence of radiographs, generic-type exercises (which do not account for actual cervical spine alignment) could worsen and further complicate abnormal spine configurations that exhibit abnormal coupling patterns due to injury, abnormal morphology, or other important differential diagnoses for neck pain. In an exercise study using primarily CVA, Goo et al. [44] state the following: “we could not investigate the effects of clinical relevance such as pain reduction, functional improvement, and improved quality of life because only people with asymptomatic mild FHP were enrolled. Second, the study focusing on reducing the CVA angle without using X-rays resulted in not being able to determine whether it was effective for cervical spine alignment accurately.”

The issue with the prescription of postural exercises in the absence of radiography or dynamic radiography, such as flexion/extension radiographs, is the high likelihood of the posture not “matching” the expected spine configuration. Patients presenting with neck pain have a high likelihood of having experienced previous neck injuries or trauma, both microtrauma from poor ergonomics and macrotrauma from motor vehicle collisions (MVCs), falls, sports injuries, etc. These injuries cause spinal structural bucking in the coronal and sagittal planes. This buckling can create first-, second-, and third-order and greater configurations, and these abnormal spine shapes can be present and impossible to visualize with external posture photographs alone [45,46,47].

**Figure 9:**
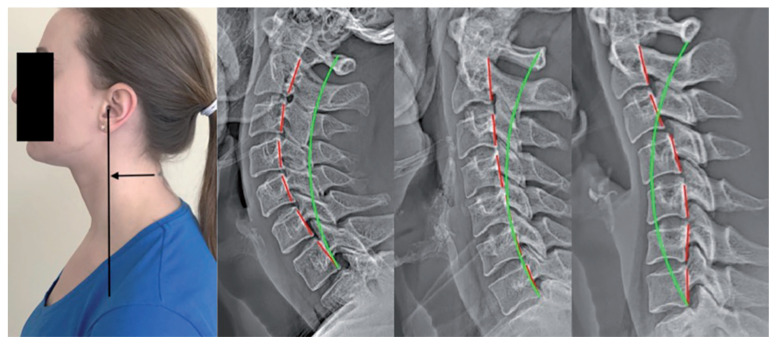
Forward head posture as shown in a posture photograph and three unique lateral cervical radiographs. All three X-ray images have about 25 mm of forward head translation, as measured with the C2–C7 SVA. Left radiograph: hyperlordosis; middle radiograph: hypolordosis; and right radiograph: kyphosis. The green line is a standard alignment; the red line highlights the patient’s alignment along the posterior body margins of cervical vertebra C2 through C7.

A classic demonstration of the “matching” versus “mismatching” of rotations and translations of posture and spine coupling patterns can be illustrated with FHP, that is, anterior head translation (Figure 9). The natural and expected spine coupling with a forward-translated head posture involves lower cervical spine flexion and upper cervical spine extension. As seen in Figure 9, many different spine vertebral coupling patterns are possible, including hyperlordosis, hypolordosis, or kyphosis, and, accordingly, each cervical configuration requires its own unique application of treatment methods for its ideal correction [47].

### 4.2. Pros and Cons of Radiographic ARA C2–C7 and C2–C7 SVA

Spine injuries, spine pain, and spine-associated conditions have been assessed via radiography for over 100 years, and, nowadays, radiography continues to play a critical part in spine assessment and treatment [10,40,42]. Radiograph acquisition requirements vary from country to country and state to state in the USA. Several states require no licensure for obtaining an X-ray, while others require certifications, licensure, or the direct observation of the image acquisition procedure by a licensed physician. Many states and countries regulate the ownership of imaging facilities, and protections are put in place for patient and operator safety. Radiography technicians are a sub-category of extended limited-scope practitioners and are able to acquire and in some cases analyze radiographs depending on the state’s regulations [48,49]. The financial investment, requirement for a special certification, and other regulations may explain some of the reasons why physicians, chiropractors, physical therapists, and other spine treatment providers do not choose to use radiography. Considering the importance of radiography for a comprehensive biomechanical analysis of the spine, it is not recommended that providers who treat patients with spinal injuries and pain make diagnoses nor treatment recommendations based on postural image assessment alone, as the current findings as well as those in other studies demonstrate this approach to be unreliable for cervical spine parameter analysis [16,39]. Additionally, the benefits of radiographic spine parameter analysis far outweigh any risks when considering the tremendous burden that neck disease and disorders represent globally; that is, radiographic screening is more informative in reaching a diagnosis.

The reliability in measuring the plumb line SVA in mm on lateral full-spine radiographs has previously been established [50,51,52]. The locally measured C2–C7 cervical (c) SVA has been previously established as being reliable for FHP and has been computed for vertical plumb line assessment compared to a standard [53,54]. Marques et al. [54] found cervical SVA to have an excellent inter-examiner correlation coefficient of 0.978, demonstrating a very high reliability in measurements. Computer programs using artificial intelligence, compared to human measurement, have demonstrated a high reliability in their measured SVA [55,56,57]. Importantly, Kato et al. found, in 2017, that “patients with a C2–7 SVA of ≥35 mm experienced severe postoperative neck pain (axial pain). The C2–7 SVA is a parameter worth considering because it can lead to poor QOL and axial neck pain after laminoplasty” [57].

Other studies have determined pathological limits for the parameter of the C2–C7 SVA. Harrison et al., in 1996, found that 15 mm of C2–C7 represented the mean forward sagittal balance in asymptomatic participants and suggested that increases in this distance would abnormally load the cervical spine and extraspinal tissues [12]. Roguski et al. found that a forward cSVA greater than 20 mm was an initial cutoff for poor outcomes and that >40 mm was strongly associated with poor outcomes and increased post-surgical complications [29]. In general, poor surgical outcomes have been reported from C2–C7 SVA > 35 mm [57], and severe pain, disability, and poor surgical outcomes have been found for SVA > 40 mm [10,14,29,57,58], while improvements in C2–C7 SVA have been correlated with positive outcomes following surgical intervention [10,58,59].

Radiography is not only necessary to determine the correct C2–C7 SVA assessment, but it is also the gold standard for cervical lordosis measurements. In 1996, Harrison et al. published a mathematical model of standard cervical lordosis and found that the cervical spine is best represented by a circular model from C1-T1 or C2–C7, the latter measuring 34° on average and 42°, in an ideal scenario [12]. In 2004, Harrison et al. further investigated the standard cervical spine lordosis parameters to discriminate between typical controls and patients with neck pain: patients with ARA C2–C7 < 20° were statistically identified as sufferers of chronic neck pain [60]. McAviney et al. confirmed that the typical limits for pain compared to patients without pain can be distinguished by a cutoff value of less than 20° for cervical lordosis as measured with the ARA C2–C7 [61]. Additionally, randomized trials have demonstrated that a 20° cervical spine lordosis is a cutoff parameter for improved patient outcomes [18,19,47].

In Figure 6, it can be readily seen that a patient with a CVA < 50° might have a C2–C7 SVA > 20 mm, and, in this example, both measurements are outside the standard limits for FHP measurements, indicating a significant amount of clinically relevant FHP. Here, using either or both an external posture measurement and a lateral cervical radiograph for the clinical decision process would indicate the same finding, and, thus, the clinical interventions would be consistent for both the posture and the X-ray. However, given the weak correlation identified in the current study, a likely scenario in Figure 6 would involve a patient whose CVA is below the standard range (<50°), who would present with a lateral cervical spine radiographic measurement of C2–C7 SVA, within the standard limits (<20 mm), indicating no FHP according to the radiography. Here, the clinical decision process would indicate contrasting findings, and, thus, the clinical diagnosis and interventions would be inconsistent for the posture and the X-ray measurement of FHP. A third scenario, considering Figure 6, would be a representative patient whose CVA is within the standard range (>55°), indicating no significant FHP during a posture assessment. In contrast, the lateral cervical spine radiographic measurement of C2–C7 SVA might be above the standard limits (>20 mm), indicating considerable FHP via radiography. Again, in this example, the clinical decision process would indicate contrasting findings, and, thus, the clinical diagnosis and interventions would be inconsistent for the posture and the X-ray measurement of FHP. These scenarios have only considered the congruency of FHP between the CVA and radiographically measured cSVA; however, since the CVA does not correlate with the lordosis of the cervical spine, it does not matter if the CVA and cSVA are congruent, as treatment considerations have been shown to be more based on the lordosis magnitude and pattern (i.e., harmonic) than just on whether there is a presence of FHP [47]. Thus, given that the photographic CVA is not able to accurately determine ARA C2–C7 or the C2–C7 SVA, it is not recommended for clinicians to diagnose nor recommend treatment without measuring lateral cervical radiographs.

### 4.3. Risk–Benefit Ratio of Exposure to Ionizing Radiation

As discussed, the low shared variance between the CVA and X-ray based C2–C7 SVA, and the important clinical consideration that the CVA does not relate to the cervical lordosis drives the obvious conclusion that for clinical assessment regarding treatment considerations, both CVA postural analysis and radiographic analysis is always preferred as it is much more precise for diagnosis and treatment recommendations. Thus, the risk–benefit aspects related to radiography must be considered. Here, we briefly debate that this is a non-issue. A recent systematic review [62] of articles published from 1975 to 2017 examined cancer risk from external low-dose X-ray and gamma radiation (<200 mSv) and assessed the higher-quality studies that support or question the role of low-dose radiation in oncogenesis. From the 4382 articles initially located, 62 articles met all the inclusion/exclusion criteria. After assessing the methodological rigor, only 25 studies met the higher-quality criteria; 21 out of the 25 studies did not support cancer induction by low-dose radiation (*p* = 0.0003) [62]. Thus, a lateral cervical X-ray (<1 mSv) is less than 1/200th of the amount of radiation reported from this rigorous review to be safe; therefore, the acquisition of a cervical radiograph will always have a beneficial risk–benefit ratio [62].

### 4.4. Limitations

As with all investigations, our study has some limitations, each of which lend themselves to a future investigation. We used a sample of convenience from one outpatient clinic, which may not be representative of the entire population of patients with CMP or other types of spine deformities. Secondly, due to the inclusion criteria, our population of patients did not have C2–C7 SVA of less than 15 mm, and there are no data available to draw comparisons to populations with a magnitude under 15 mm. Likewise, our population of patients did not have CVA >50° (one patient had a 51° CVA), so it is unknown how populations with a CVA greater than 50° might correlate to their radiographically measured C2–C7 SVA. Along this line of thought, our population did not have large magnitudes of radiographic C2–C7 SVA displacements, as the largest displacement was found to be 37 mm; it is possible that, in larger displacements, there would be a stronger correlation between postural photographic CVA and radiographic C2–C7 SVA measurements. Regarding cervical lordosis (ARA C2–C7), we did not investigate cervical kyphotic curvatures, so it is unknown how these types of curvatures might correlate to the CVA. Finally, it is possible that other photographic measurements of FHP (plumbline, etc.) might have stronger correlations to the radiographic measurement of C2–C7 SVA.

Finally, regarding the measurement methods and statistical analysis used herein, it might be argued that the measurement methods we used lack sensitivity and reliability to accurately quantify the FHP externally (CVA) and internally with spine radiography. However, both the CVA [16,17] and the radiographic methods [11,16,26] employed in this investigation have excellent examiner reliability and validity for the variable they are assessing. Similarly, our choice of the regression analysis using the R^2^ value is the appropriate method to assess the strength of the relationship between a dependent variable and an independent one, where R^2^ explains the extent of variation in the first variable that is driven by the second variable [30]. Lastly, our sample size was more than adequate to assess the correlation between the CVA and the X-ray-measured variables.

## 5. Conclusions

In rehabilitation settings, postural measurements are emphasized, while, in the surgical setting, radiography is favored; this may create conflicting understandings, interventions, and outcomes. In a prospective sample of 120 participants with CMP pain syndrome, we hypothesized that the CVA externally would show a moderate-to-strong correlation to radiographic measurements of C2–C7 SVA and lordosis. In contrast, this study demonstrated a weak correlation between the CVA external posture measurement and the internal radiographic measurement (C2–C7 SVA) for the assessment of forward head posture, as well as the CVA and the radiographic measurement for the assessment of cervical lordosis (ARA C2–C7). In some cases, the CVA undervalued the C2–C7 SVA, while, in others, it overvalued it. Importantly, the CVA cannot be used to predict the amount of cervical lordosis (ARA C2–C7) in a given patient. While these two measurements of FHP are weakly correlated, they are quite different in their distribution in an individual and may be representing different aspects of sagittal cervical balance. Clinically, an over-reliance on external postural measures may lead to mismanagement in terms of postural rehabilitation; thus, we recommend that more emphasis be given to the C2–C7 SVA and ARA C2–C7 cervical lordosis on cervical radiographs versus the CVA when patient interventions and outcomes are at stake. Understanding the significant difference between external neck posture and the actual spine alignment inside of the body is crucial in understanding the evaluation, diagnosis, treatment, and long-term outlook for spine interventions.

## Figures and Tables

**Table 1:** Descriptive data for the demographic variables for 120 patients. The values are presented as the mean and standard deviation (SD) for all the variables except gender (%).

Variable	Mean ± SD		
Age (years)	32.5 ± 7.5		
Weight (kg)	77 ± 10.5		
Neck pain intensity (NRS)	5.2 ± 0.8		
**Gender (%)**			
Male	63.3		
Female	36.7		
**FHP Variables**	**Mean ± SD**	**Maximum**	**Minimum**
AHT Tz C2–C7 (cm)	2.46 ± 0.62	3.7	1.5
ARA C2–C7 (°)	−6.80 ± 5.98	−17	0
CVA (°)	44.9 ± 3.33	51	40

Note: AHT Tz C2–C7: anterior head translation; NRS: numerical rating scale 0–10; and CVA: craniovertebral angle. A negative value (−) indicates cervical extension.

## Data Availability

Additional pertinent data are available upon request.

## Abbreviations

AHT	Anterior Head Translation
ARA	Absolute Rotation Angle
CVA	Craniovertebral Angle
CMP	Chronic Myofascial Pain
FHP	Forward Head Posture
ICC	Intra-examiner Correlation Coefficient
ROM	Range Of Motion
RRA	Relative Rotation Angle
SVA	Sagittal Vertical Axis
